# Associations of mutually exclusive categories of physical activity and sedentary time with markers of cardiometabolic health in English adults: a cross-sectional analysis of the Health Survey for England

**DOI:** 10.1186/s12889-016-2694-9

**Published:** 2016-01-12

**Authors:** Kishan Bakrania, Charlotte L. Edwardson, Danielle H. Bodicoat, Dale W. Esliger, Jason M. R. Gill, Aadil Kazi, Latha Velayudhan, Alan J. Sinclair, Naveed Sattar, Stuart J. H. Biddle, Kamlesh Khunti, Melanie Davies, Thomas Yates

**Affiliations:** 1 Department of Health Sciences, University of Leicester, Leicester Diabetes Centre, Leicester General Hospital, Gwendolen Road, Leicester, LE5 4PW United Kingdom; 2 Diabetes Research Centre, University of Leicester, Leicester Diabetes Centre, Leicester General Hospital, Gwendolen Road, Leicester, LE5 4PW UK; 3 National Institute for Health Research (NIHR) Leicester-Loughborough Diet, Lifestyle and Physical Activity Biomedical Research Unit (BRU), Leicester Diabetes Centre, Leicester General Hospital, Gwendolen Road, Leicester, LE5 4PW UK; 4 School of Sport, Exercise and Health Sciences, Loughborough University, Loughborough, LE11 3TU UK; 5 British Heart Foundation Glasgow Cardiovascular Research Centre (BHF GCRC), Institute of Cardiovascular and Medical Sciences, College of Medical, Veterinary and Life Sciences, University of Glasgow, Glasgow, G12 8TA UK; 6 Psychiatry for the Elderly, Department of Health Sciences, University of Leicester, Leicester, LE1 7RH UK; 7 Diabetes Frail Ltd, University of Aston, Birmingham, B4 7ET UK; 8 Institute of Sport, Exercise & Active Living, Victoria University, Melbourne, Australia; 9 National Institute for Health Research (NIHR) Collaboration for Leadership in Applied Health Research and Care – East Midlands (CLAHRC – EM) Leicester Diabetes Centre, Leicester General Hospital, Gwendolen Road, Leicester, LE5 4PW UK

**Keywords:** Accelerometry, Objective, Physical activity, Sedentary, Cardiometabolic health

## Abstract

**Background:**

Both physical activity and sedentary behaviour have been individually associated with health, however, the extent to which the combination of these behaviours influence health is less well-known. The aim of this study was to examine the associations of four mutually exclusive categories of objectively measured physical activity and sedentary time on markers of cardiometabolic health in a nationally representative sample of English adults.

**Methods:**

Using the 2008 Health Survey for England dataset, 2131 participants aged ≥18 years, who provided valid accelerometry data, were included for analysis and grouped into one of four behavioural categories: (1) ‘Busy Bees’: physically active & low sedentary, (2) ‘Sedentary Exercisers’: physically active & high sedentary, (3) ‘Light Movers’: physically inactive & low sedentary, and (4) ‘Couch Potatoes’: physically inactive & high sedentary. ‘Physically active’ was defined as accumulating at least 150 min of moderate-to-vigorous physical activity (MVPA) per week. ‘Low sedentary’ was defined as residing in the lowest quartile of the ratio between the average sedentary time and the average light-intensity physical activity time. Weighted multiple linear regression models, adjusting for measured confounders, investigated the differences in markers of health across the derived behavioural categories. The associations between continuous measures of physical activity and sedentary levels with markers of health were also explored, as well as a number of sensitivity analyses.

**Results:**

In comparison to ‘Couch Potatoes’, ‘Busy Bees’ [body mass index: −1.67 kg/m^2^ (*p* < 0.001); waist circumference: −1.17 cm (*p* = 0.007); glycated haemoglobin: −0.12 % (*p* = 0.003); HDL-cholesterol: 0.09 mmol/L (*p* = 0.001)], ‘Sedentary Exercisers’ [body mass index: −1.64 kg/m^2^ (*p* < 0.001); glycated haemoglobin: −0.11 % (*p* = 0.009); HDL-cholesterol: 0.07 mmol/L (*p* < 0.001)] and ‘Light Movers’ [HDL-cholesterol: 0.11 mmol/L (*p* = 0.004)] had more favourable health markers. The continuous analyses showed consistency with the categorical analyses and the sensitivity analyses indicated robustness and stability.

**Conclusions:**

In this national sample of English adults, being physically active was associated with a better health profile, even in those with concomitant high sedentary time. Low sedentary time independent of physical activity had a positive association with HDL-cholesterol.

**Electronic supplementary material:**

The online version of this article (doi:10.1186/s12889-016-2694-9) contains supplementary material, which is available to authorized users.

## Background

There is increasing evidence that sedentary behaviour, characterised as any waking behaviour with low energy expenditure (≤1.5 metabolic equivalents) while in a sitting or reclining posture [1], is strongly associated with a number of health outcomes [2–8]. These studies have shown that high levels of sedentary behaviour are associated with a greater risk of morbidity and mortality, which is potentially concerning given that most adults spend the majority of their waking hours (~55 % to ~70 %) engaging in this behaviour [9–12]. In contrast, it is known that high levels of physical activity, particularly moderate-to-vigorous physical activity (MVPA), are associated with improved health, often in a dose–response manner [13–15]. Sedentary behaviour and MVPA share a weak inverse relationship and it is possible for an individual, over the course of a day, to have high levels of physical activity and still accumulate large amounts of sedentary time [16–18].

Previous research has largely focused on the independent associations of total physical activity, MVPA, light-intensity physical activity and sedentary time on health [2–8, 16–27], rather than the interplay between these constructs. As a consequence, the daily equilibrium between physical activity and sedentary behaviour, and the pooled relationship they share with biomarkers of health, is not fully understood. Although some studies have started to explore different techniques for quantifying combined connections and patterns of MVPA and sedentary behaviour [28–35], to our knowledge, only one study based in the USA has investigated the associations between categories of physical activity and sedentary time with markers of health [36]. Loprinzi and colleagues found that in comparison to adults who engaged in <150 min/week of MVPA with high sedentary time (sedentary time > light-intensity physical activity time), participants engaging in ≥150 min/week of MVPA had a more favourable cardiometabolic health profile regardless of their sedentary status [36], suggesting that regular MVPA may offset some of the harmful consequences of a habitually sedentary lifestyle. If verified, this would be a clinically important message for a large proportion of the population who may be concerned about the amount of time they spend sitting.

The aim of this paper is to use the 2008 Health Survey for England (HSE) [11, 37] dataset to examine and quantify the combined categories of objectively measured physical activity and sedentary time amongst English adults and associate these factors to clinically relevant anthropometric and biochemical markers of cardiometabolic health.

## Methods

### Study sample

The HSE is a series of national annual surveys designed to examine the health and well-being of people living in England [11, 37]. In order to obtain a population-based sample, these cross-sectional surveys employ a multistage stratified random sampling procedure with postcode regions acting as the primary sampling unit. The 2008 wave was centred on physical activity and fitness and sampled 22623 participants [aged ≥2 years]. Six thousand two hundred and fourteen individuals [aged ≥4 years] were randomly selected and approached to wear an accelerometer. Adults [aged ≥18 years] who had accelerometry data available were included in the present study (*n* = 2313). Participants provided written informed consent. Ethical approval for the 2008 HSE survey was obtained from the Oxford A Research Ethics Committee (reference number 07/H0604/102). Further details regarding this sample can be found elsewhere [11, 37].

### Measuring physical activity and sedentary time

Physical activity and sedentary time were measured using an ActiGraph GT1M accelerometer (ActiGraph Corporation, Pensacola, Florida, USA) worn on the right hip for seven consecutive days during waking hours (except water-based activities) [11]. The ActiGraph GT1M device was initialised to collect data using one minute epochs. Accelerometer files were processed using KineSoft V3.3.76 (KineSoft, Loughborough, UK). Accelerometer counts were used to calculate the time spent in each intensity band: sedentary behaviour (<100 counts per min (cpm)), light-intensity physical activity (100–1951 cpm) and MVPA (≥1952 cpm) [38]. In addition, MVPA time accumulated in bouts of ≥10 min, allowing for a two minute exception in the intensity threshold, was also derived. Non-wear time was defined as any periods of continuous zero counts for ≥60 consecutive min [39]. A valid day was defined as ≥10 hours (i.e. ≥600 min) of wear-time. Adults who provided ≥4 days of valid accelerometer data were included.

### Derivation of the behavioural categories

**Fig. 1:**
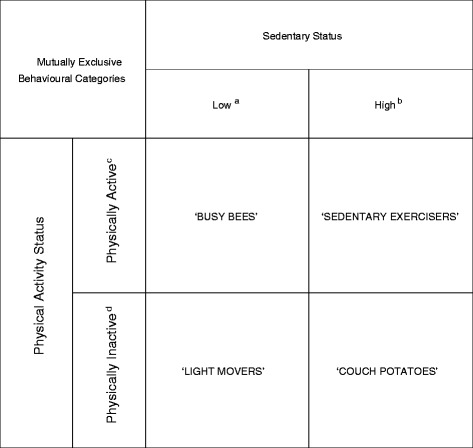
Mutually exclusive behavioural categories. ‘Busy Bees’: Physically Active and Low Sedentary, ‘Sedentary Exercisers’: Physically Active and High Sedentary, ‘Light Movers’: Physically Inactive and Low Sedentary, ‘Couch Potatoes’: Physically Inactive and High Sedentary. ^a^ Low Sedentary: Quartile 1 of the ratio between the average sedentary time and the average light-intensity physical activity time. ^b^ High Sedentary: Quartiles 2, 3 or 4 of the ratio between the average sedentary time and the average light-intensity physical activity time. ^c^ Physically Active: ≥150 min of moderate-to-vigorous physical activity per week. ^d^ Physically Inactive: <150 min of moderate-to-vigorous physical activity per week

For each individual, the average number of minutes per valid day spent in MVPA, light-intensity physical activity and sedentary behaviour were calculated. Based upon other studies [36, 40], the sedentary behaviour-to-light-intensity physical activity ratio (average sedentary time ÷ average light-intensity physical activity time) was used for the classification of sedentary status. Participants were then split into quartiles based on this ratio. Given that the levels of sedentary behaviour in the general population are predominantly high [9–12], a conservative, data-driven approach was undertaken and individuals were classified as ‘low sedentary’ if they resided in quartile 1 and ‘high sedentary’ if they resided in quartiles 2, 3 or 4. MVPA status was classified as ‘physically active’ or ‘physically inactive’ on the basis of whether or not participants accumulated at least 150 min of MVPA per week. This allowed the formation of four mutually exclusive behavioural categories (Fig. 1), which are provided with communicative names to aid interpretability: (1) ‘Busy Bees’: physically active & low sedentary, (2) ‘Sedentary Exercisers’: physically active & high sedentary, (3) ‘Light Movers’: physically inactive & low sedentary, and (4) ‘Couch Potatoes’: physically inactive & high sedentary.

### Markers of cardiometabolic health

A trained interviewer recorded height (measured to the nearest 0.1 cm using a portable stadiometer) and weight (measured to the nearest 0.1 kg using an electronic scale) readings [37]. Body Mass Index (BMI) was calculated as the weight (in kilograms) divided by the square of the height (in metres). Waist circumference was defined as the midpoint between the lower rib and the upper boundary of the iliac crest. A nurse measured this twice to the nearest 0.1 cm using a tape and the mean of the two readings was used [37]. Non-fasting blood samples were collected by the nurse for the analysis of high-density lipoprotein (HDL) cholesterol, total cholesterol and glycated haemoglobin (HbA1c). Blood analytes were assayed at the Royal Victoria Infirmary laboratory in Newcastle upon Tyne, England. Further details regarding these variables can be found elsewhere [37].

### Contextual variables

The following factors, collected by a trained interviewer [37], were also utilised: age (in years); cardiovascular disease index (no cardiovascular diseases, one or more cardiovascular diseases); fruit and vegetable consumption (0, 1–3, 4–6, 7+ portions per day); ethnicity (white, non-white); sex (male, female); smoking status (never smoked, ex-smoker, current smoker); socioeconomic status (national statistics socioeconomic classification: high, high-intermediate, intermediate, low-intermediate, low); blood pressure medication (no, yes); cholesterol medication (no, yes); and any other prescribed medication (no, yes). The ‘cardiovascular disease index’ variable was based on the following physician diagnosed cardiovascular conditions/events: abnormal heart rhythm; angina; atrial fibrillation; congenital heart disease; heart attack; heart transplant; heart valve disease; intermittent claudication; stroke; and transient ischaemic attack.

### Statistical analysis

All statistical analyses were conducted using Stata/IC V13.1 (Stata Corporation, College Station, Texas, USA) and controlled for the complex survey strategy employed in the 2008 HSE (primary sampling units, clustering and survey weights) [11, 37]. Interview weights, which adjusted for: household selection; non-response bias; age; sex; and regional profiles, were applied in order to produce estimates representing the national population. Nurse weights (generated from interview weights) were utilised to further reduce non-response bias arising from individuals who were interviewed but did not have a nurse visit. Blood weights (generated from nurse weights) were utilised to analyse the blood related variables.

### Categorical associations

Pairwise deletion was used throughout the study for handling any missing data. The weighted prevalence [n; %] of the English adults in each mutually exclusive behavioural category were computed. Participant characteristics of the full sample, stratified by each category, were tabulated. Categorical variables were presented as proportions [n; %], whereas the continuous variables were summarised via their means and standard errors. Univariate linear regression models, with ‘behavioural category’ as the independent variable, were fitted for the following assessed health markers: BMI; waist circumference; HDL-cholesterol; total cholesterol; and HbA1c. The ‘Couch Potatoes’ category, representing physically inactive adults with high sedentary time (i.e. the least desirable group), was selected as the reference category. Subsequently, multiple linear regression models were also fitted for each dependent variable with the following covariates: age; BMI (except in the model with BMI as the dependent variable); cardiovascular disease index; ethnicity; fruit and vegetable consumption; sex; smoking status; socioeconomic status; and accelerometer wear-time. Models with HDL-cholesterol and total cholesterol as the dependent variable were also controlled for both blood pressure medication and cholesterol medication. Similarly, the model with HbA1c as the dependent variable was controlled for any prescribed medication. All regression analyses were two-sided where *p* < 0.01 was considered to be statistically significant to account for multiple comparisons.

The following sensitivity analyses, examining the robustness of the main effects, were also investigated: (1) missing data in the covariates were imputed using the behavioural category means (continuous variables: BMI) and modes (categorical variables: smoking status and socioeconomic status), (2) participants with a cardiovascular disease index of ‘one or more cardiovascular diseases’ were excluded, (3) ‘Low Sedentary’ was defined as residing in the lowest tertile of the ratio between the average sedentary time and the average light-intensity physical activity time, and (4) participants were only classified into the ‘physically active’ categories if they accumulated ≥150 min of MVPA per week in bouts of ≥10 min. For each sensitivity analysis, the weighted behavioural category prevalence and health associations were reported.

### Continuous associations

The associations between continuous measures of physical activity and sedentary levels with markers of health were also investigated. Multiple linear regression models were fitted for each health marker whilst controlling for the appropriate corresponding confounders as well as both continuous MVPA time and sedentary status (sedentary-behaviour-to-light-intensity physical activity ratio). Analogous to the categorical analyses, these regression analyses were also two-sided with *p* < 0.01 considered to be statistically significant.

## Results

A total of 2131 adults were available for analysis after retaining only those individuals who provided ≥4 valid days of accelerometer data. The four groups were comprised as follows: (1) ‘Busy Bees’: *n* = 385; 18.6 %, (2) ‘Sedentary Exercisers’: *n* = 743; 36.7 %, (3) ‘Light Movers’: *n* = 147; 6.8 %, and (4) ‘Couch Potatoes’: *n* = 856; 37.9 %. Table 1 presents the characteristics of the participants.

**Table 1:** Participant characteristics

Characteristic	Sample	‘Busy Bees’	‘Sedentary Exercisers’	‘Light Movers’	‘Couch Potatoes’
	*N* = 2131	*n* = 385; 18.6 %	*n* = 743; 36.7 %	*n* = 147; 6.8 %	*n* = 856; 37.9 %
Age (in years)^a^	50.8 (0.47)	44.8 (0.72)	45.6 (0.72)	49.8 (1.27)	58.9 (0.74)
Cardiovascular Disease Index^b^					
No Cardiovascular Diseases	2030 (95.6)	374 (97.4)	707 (95.7)	141 (96.2)	808 (94.6)
One or More Cardiovascular Diseases	101 (4.4)	11 (2.6)	36 (4.3)	6 (3.8)	48 (5.4)
Ethnicity^b^					
White	2008 (93.2)	364 (93.6)	691 (92.1)	141 (95.1)	812 (93.8)
Non-White	123 (6.8)	21 (6.4)	52 (7.9)	6 (4.9)	44 (6.2)
Fruit & Vegetable Consumption (portions per day)^b^					
0	95 (4.5)	25 (6.1)	29 (3.9)	8 (5.5)	33 (4.0)
1–3	680 (32.4)	128 (34.8)	221 (30.6)	46 (30.9)	285 (33.4)
4–6	968 (45.3)	152 (39.0)	359 (48.0)	69 (48.4)	388 (45.3)
7+	388 (17.8)	80 (20.1)	134 (17.5)	24 (15.2)	150 (17.3)
Sex^b^					
Male	981 (49.3)	172 (48.8)	414 (59.0)	36 (28.8)	359 (43.8)
Female	1150 (50.7)	213 (51.2)	329 (41.0)	111 (71.2)	497 (56.2)
Smoking Status^b^					
Never Smoked	993 (47.1)	171 (44.5)	393 (53.7)	57 (37.8)	372 (43.6)
Ex-Smoker	726 (32.7)	113 (28.6)	232 (29.5)	51 (34.0)	330 (37.5)
Current Smoker	410 (20.1)	101 (26.9)	116 (16.4)	39 (28.2)	154 (18.9)
Missing^c^	2 (0.1)	0 (0.0)	2 (0.4)	0 (0.0)	0 (0.0)
Socioeconomic Status^b^					
High	769 (36.7)	80 (20.5)	358 (49.0)	34 (22.5)	297 (35.1)
High-Intermediate	276 (12.4)	41 (9.6)	100 (13.2)	22 (13.4)	113 (12.8)
Intermediate	203 (9.5)	48 (12.4)	48 (6.2)	18 (13.7)	89 (10.5)
Low-Intermediate	191 (9.3)	52 (15.0)	44 (5.8)	14 (11.4)	81 (9.6)
Low	646 (29.8)	154 (40.1)	177 (23.3)	58 (38.5)	257 (29.5)
Missing^c^	46 (2.3)	10 (2.4)	16 (2.5)	1 (0.5)	19 (2.5)
Blood Pressure Medication^b^					
No	1629 (78.7)	349 (91.1)	636 (87.6)	119 (82.5)	525 (63.4)
Yes	502 (21.3)	36 (8.9)	107 (12.4)	28 (17.5)	331 (36.6)
Cholesterol Medication^b^					
No	1797 (85.8)	366 (95.7)	669 (91.3)	129 (88.5)	633 (75.2)
Yes	334 (14.2)	19 (4.3)	74 (8.7)	18 (11.5)	223 (24.8)
Any Prescribed Medication^b^					
No	1031 (51.2)	238 (63.2)	426 (60.1)	80 (57.4)	287 (35.5)
Yes	1100 (48.8)	147 (36.8)	317 (39.9)	67 (42.6)	569 (64.5)
Moderate-to-Vigorous Physical Activity Time^a^ (no. of minutes per valid day)	30.3 (0.59)	51.3 (1.46)	44.0 (0.80)	13.2 (0.39)	9.7 (0.22)
Sedentary Time ^a^ (no. of minutes per valid day)	540.2 (2.29)	417.2 (3.62)	564.5 (2.68)	435.5 (3.84)	595.7 (2.39)
Light-Intensity Physical Activity Time^a^ (no. of minutes per valid day)	289.1 (2.06)	394.9 (3.16)	260.5 (1.88)	396.2 (4.35)	245.9 (2.47)
Accelerometer Wear-Time^a^ (no. of minutes per valid day)	859.7 (1.72)	863.5 (4.53)	869.1 (2.69)	844.9 (5.65)	851.4 (2.78)
Sedentary-to-Light-Intensity Physical Activity Time Ratio^a^	2.2 (0.03)	1.1 (0.01)	2.3 (0.03)	1.1 (0.01)	2.8 (0.05)
Number of Valid Days^b^					
4	99 (4.9)	18 (4.7)	35 (5.1)	8 (5.7)	38 (4.8)
5	185 (9.1)	36 (9.7)	55 (8.1)	21 (14.6)	73 (8.8)
6	414 (20.4)	80 (21.0)	139 (20.1)	32 (23.5)	163 (20.0)
7	1433 (65.6)	251 (64.6)	514 (66.7)	86 (56.2)	582 (66.4)
Body Mass Index (kg/m^2^)^a^	27.5 (0.12)	26.6 (0.24)	26.8 (0.17)	27.6 (0.43)	28.7 (0.21)
Missing^c^	185 (8.7)	30 (7.8)	44 (5.9)	8 (5.4)	103 (12.0)
Waist Circumference (cm)^a^	93.4 (0.36)	90.1 (0.70)	91.7 (0.57)	91.5 (1.18)	97.0 (0.55)
Missing^c^	242 (11.4)	44 (11.4)	80 (10.8)	17 (11.6)	101 (11.8)
HDL-Cholesterol (mmol/L)^a^	1.49 (0.01)	1.53 (0.02)	1.49 (0.02)	1.58 (0.04)	1.44 (0.01)
Missing^c^	728 (34.2)	120 (31.2)	245 (33.0)	51 (34.7)	312 (36.4)
Total Cholesterol (mmol/L)^a^	5.42 (0.03)	5.37 (0.06)	5.49 (0.05)	5.48 (0.12)	5.37 (0.05)
Missing^c^	728 (34.2)	120 (31.2)	245 (33.0)	51 (34.7)	312 (36.4)
Glycated Haemoglobin (%)^a^	5.64 (0.02)	5.47 (0.02)	5.51 (0.02)	5.89 (0.14)	5.82 (0.04)
Missing^c^	746 (35.0)	127 (33.0)	250 (33.6)	52 (35.4)	317 (37.0)

All analyses accounted for primary sampling units, clustering and survey weights

*‘Busy Bees’*: Physically Active and Low Sedentary, *‘Sedentary Exercisers’*: Physically Active and High Sedentary, *‘Light Movers’*: Physically Inactive and Low Sedentary, *‘Couch Potatoes’*: Physically Inactive and High Sedentary

^a^Continuous variable; Mean (Standard Error)

^b^Categorical variable; *n* (Proportion (%))

^c^Missing data; n (%)

### Categorical associations

The unadjusted and adjusted categorical analyses are displayed in Table 2 and Additional file 1: Figure S1. The adjusted analyses showed that in comparison to ‘Couch Potatoes’, ‘Busy Bees’ had significantly lower BMI (*p* < 0.001), waist circumference (*p* = 0.007) and HbA1c (*p* = 0.003) levels, and higher HDL-cholesterol (*p* = 0.001) levels. Similarly, ‘Sedentary Exercisers’ had significantly lower BMI (*p* < 0.001) and HbA1c (*p* = 0.009) levels, and higher HDL-cholesterol (*p* < 0.001) levels. ‘Light Movers’ had significantly higher HDL-cholesterol (*p* = 0.004) levels.

**Table 2:** Categorical associations with markers of cardiometabolic health (beta coefficients (99% CIs) and corresponding *p*-values)

Health marker	Linear regression model	‘Busy Bees’		‘Sedentary Exercisers’		‘Light Movers’		‘Couch Potatoes’
		Beta (99% CI)	*p*-value	Beta (99% CI)	*p*-value	Beta (99% CI)	*p*-value	
Body Mass Index (kg/m^2^)	Unadjusted	**−2.06 (−2.86, −1.26)**	**<0.001**	**−1.93 (−2.61, −1.25)**	**<0.001**	−1.04 (−2.26, 0.18)	0.027	Reference
	Adjusted	**−1.67 (−2.57, −0.77)**	**<0.001**	**−1.64 (−2.43, −0.85)**	**<0.001**	−0.66 (−1.92, 0.60)	0.175	Reference
Waist Circumference (cm)	Unadjusted	**−6.92 (−9.17, −4.68)**	**<0.001**	**−5.31 (−7.33, −3.30)**	**<0.001**	**−5.53 (−8.89, −2.16)**	**<0.001**	Reference
	Adjusted	**−1.17 (−2.28, −0.06)**	**0.007**	−0.71 (−1.56, 0.14)	0.032	−0.07 (−1.61, 1.47)	0.908	Reference
HDL-Cholesterol (mmol/L)	Unadjusted	**0.09 (0.02, 0.17)**	**0.001**	0.05 (−0.01, 0.11)	0.021	**0.14 (0.04, 0.24)**	**<0.001**	Reference
	Adjusted	**0.09 (0.02, 0.16)**	**0.001**	**0.07 (0.02, 0.13)**	**<0.001**	**0.11 (0.01, 0.21)**	**0.004**	Reference
Total Cholesterol (mmol/L)	Unadjusted	−0.00 (−0.21, 0.21)	0.981	0.12 (−0.06, 0.29)	0.081	0.11 (−0.23, 0.45)	0.408	Reference
	Adjusted	0.02 (−0.17, 0.22)	0.761	0.17 (−0.01, 0.35)	0.014	0.08 (−0.22, 0.38)	0.490	Reference
Glycated Haemoglobin (%)	Unadjusted	**−0.35 (−0.47, −0.24)**	**<0.001**	**−0.32 (−0.43, −0.20)**	**<0.001**	0.07 (−0.32, 0.46)	0.656	Reference
	Adjusted	**−0.12 (−0.22, −0.01)**	**0.003**	**−0.11 (−0.23, −0.01)**	**0.009**	0.26 (−0.11, 0.63)	0.072	Reference

All analyses accounted for primary sampling units, clustering and survey weights. Unadjusted and adjusted linear regression models were fitted for each cardiometabolic health marker with the ‘Couch Potatoes’ category selected as the reference group. The adjusted linear regression models controlled for: age; body mass index (except in the model with body mass index as the dependent variable); cardiovascular disease index; ethnicity; fruit and vegetable consumption; sex; smoking status; socioeconomic status; and accelerometer wear-time. Models with HDL-cholesterol and total cholesterol as the dependent variable were also controlled for both blood pressure medication and cholesterol medication. Similarly, the model with glycated haemoglobin as the dependent variable was controlled for any prescribed medication. **Bold** indicates statistical significance at α = 0.01

*‘Busy Bees’*: Physically Active and Low Sedentary, *‘Sedentary Exercisers’*: Physically Active and High Sedentary, *‘Light Movers’*: Physically Inactive and Low Sedentary, *‘Couch Potatoes’*: Physically Inactive and High Sedentary

The sensitivity analyses, including an alternative less conservative method for classifying sedentary status, indicated robustness and stability. Although the prevalence in each category varied across the different methods used (Additional file 1: Table S1), the main results from the primary multiple linear regression models were largely unaffected (Additional file 1: Table S2).

### Continuous associations

The adjusted continuous analyses are displayed in Table 3 and showed consistency with the categorical analyses. These models, which controlled for relevant confounders as well as both MVPA time and sedentary status, revealed that MVPA time was significantly associated with lower BMI (*p* < 0.001), waist circumference (*p* < 0.001) and HbA1c (*p* = 0.002) levels, and higher HDL-cholesterol (*p* < 0.001) levels. In contrast, sedentary status was significantly associated with lower HDL-cholesterol (*p* = 0.004) levels.

**Table 3:** Continuous associations with markers of cardiometabolic health (beta coefficients (99% CIs) and corresponding *p*-values)

Health marker	Moderate-to-vigorous physical activity time		Sedentary behaviour-to-light-intensity physical activity ratio	
	Beta (99 % CI)^a^	*p*-value	Beta (99 % CI)^b^	*p*-value
Body Mass Index (kg/m^2^)	**−0.0393 (−0.0505, −0.0282)**	**<0.001**	−0.1109 (−0.3918, 0.1700)	0.305
Waist Circumference (cm)	**−0.0315 (−0.0442, −0.0188)**	**<0.001**	0.1380 (−0.2502, 0.5261)	0.355
HDL-Cholesterol (mmol/L)	**0.0019 (0.0009, 0.0029)**	**<0.001**	**−0.0253 (−0.0476, −0.0030)**	**0.004**
Total Cholesterol (mmol/L)	0.0006 (−0.0024, 0.0036)	0.606	−0.0480 (−0.1335, 0.0376)	0.146
Glycated Haemoglobin (%)	**−0.0021 (−0.0037, −0.0004)**	**0.002**	−0.0079 (−0.0564, 0.0407)	0.673

All analyses accounted for primary sampling units, clustering and survey weights. Adjusted linear regression models were fitted for each cardiometabolic health marker. The models controlled for: age; body mass index (except in the model with body mass index as the dependent variable); cardiovascular disease index; ethnicity; fruit and vegetable consumption; sex; smoking status; socioeconomic status; **moderate-to-vigorous physical activity time; sedentary behaviour-to-light-intensity physical activity ratio**; and accelerometer wear-time. Models with HDL-cholesterol and total cholesterol as the dependent variable were also controlled for both blood pressure medication and cholesterol medication. Similarly, the model with glycated haemoglobin as the dependent variable was controlled for any prescribed medication. **Bold** indicates statistical significance at α = 0.01

^a^Beta coefficients represent a one minute increase in moderate-to-vigorous physical activity time per day

^b^Beta coefficients represent a one unit increase in the sedentary behaviour-to-light-intensity physical activity ratio

## Discussion

This is the first study to quantify associations of mutually exclusive categories of objectively measured physical activity and sedentary time with markers of cardiometabolic health in a nationally representative sample of English adults. Overall, adults who engaged in at least 150 min of MVPA per week, including those with concomitant high sedentary time (‘Sedentary Exercisers’), had more favourable health profiles compared to physically inactive individuals with high sedentary time (‘Couch Potatoes’). Low sedentary time independent of physical activity (‘Light Movers’) had positive associations with HDL-cholesterol. These findings were consistent with the sensitivity and continuous analyses.

The approach to categorising the population into one of four mutually exclusive categories extends previous research using HSE. For example, previous analysis of HSE has reported associations between both self-reported and objectively assessed sedentary time with markers of health in working age and older adults and between MVPA and markers of health [25–27]. The wider evidence has increasingly demonstrated that objectively measured sedentary time is independently associated with markers of cardiometabolic health [16–20, 41, 42], although not all studies have demonstrated this link [43]. Whilst these previous analyses have adjusted for MVPA, the associations of sedentary time with health across physical activity levels are less well understood. Therefore, this study adds to the evidence by investigating associations of sedentary status with health across categories of physical activity.

Our findings are in broad agreement with the only other study to have used a similar methodology [36]. Using national survey data from the USA, Loprinzi and colleagues found that in comparison to individuals (aged ≥20 years) who engaged in <150 min/week of MVPA with high sedentary time (sedentary time > light-intensity physical activity time), individuals engaging in ≥150 min/week of MVPA had a more favourable cardiometabolic profile (BMI, waist circumference, C-reactive protein, white blood cells and neutrophils) regardless of their sedentary status. [36] Participants in the most desirable group (≥150 min/week of MVPA with low sedentary time) also had better HDL-cholesterol, triglyceride and insulin levels. Similar to our study, participants in the physically inactive group with low sedentary time had fewer beneficial associations, although more favourable profiles for triglycerides and insulin levels were still observed.

Our findings, alongside those of Loprinzi and colleagues [36], are also consistent with the emerging evidence that levels of fitness or physical activity may modify the associations between sedentary time and markers of health in adults [35, 41, 44, 45], with sedentary behaviour only emerging as a determinant of health in those who are inactive or unfit. Together, these studies suggest that being physically active may confer some protection from the potentially deleterious impact of high sedentary behaviour.

In our study, low sedentary time in the absence of being physically active (‘Light Movers’) was associated with higher levels of HDL-cholesterol (+0.11 mmol/L), suggesting that physical inactivity in a combination with low sedentary time may have some positive relationships with health. However, the potential benefits appeared to be less numerous and consistent than those observed for physically active categories (‘Busy Bees’ and ‘Sedentary Exercisers’). One reason for this could be in the assessed markers of cardiometabolic health. Although our study included a measure of glycaemia (HbA1c), more sensitive measures of insulin resistance, which have shown stronger associations with sedentary time [6, 19, 20, 46], were not available.

Although sedentary behaviour and MVPA have been hypothesised to be distinctive health behaviours, it is unclear to what extent the underlying mechanisms act through the same or independent pathways. This reflects a limitation in the evidence more generally where mechanisms underpinning the benefits of sedentary behaviour have not been adequately elucidated. To date, the only evidence-based independent mechanism for sedentary behaviour has been through the activation of lipoprotein lipase which has been shown to change by a factor of 10 in animal models following hind limb suspension [47]. This supports the observation in our study where low sedentary time was associated with higher HDL-cholesterol levels, even in those who were physically inactive (‘Light Movers’). In contrast to sedentary behaviour, acute and chronic physiological adaptions have been well established linking higher levels of physical activity to cardiometabolic health [48–50].

### Strengths and limitations

Our study has several strengths and some limitations. Strengths include; exploitation of a well-characterised national survey which employs a multifaceted stratified random sampling procedure; utilising objectively measured physical activity and sedentary behaviour data; and a wide range of sensitivity analyses to test the robustness of our findings. The method used for deriving sedentary status has both strengths and limitations in itself. In contrast to Loprinzi and colleagues [36], who used a pre-defined method for classifying high/low sedentary time, we took a conservative, data-driven approach. Differences between accelerometer characteristics, such as wear-time, across populations can have a significant effect on the average sedentary time, artificially inflating or deflating the number of participants falling within a fixed threshold. For example, based on the method used by Loprinzi and colleagues [36], less than 8 % of the participants in our sample would be classified as ‘Low Sedentary’, and only 1.6 % of the population would be categorised as physically inactive with low sedentary time i.e. ‘Light Movers’ (data not shown). Therefore, the approach used in our study ensures that the categories are determined in relation to the population characteristics and not influenced by measurement artefact. However, the corresponding limitation is that specific targets for intervention are difficult to define. Additional pertinent limitations that are applicable to our study include the cross-sectional design which prohibits the possibility of establishing causality (reverse-causality remains open) or that unmeasured variables were confounding observed associations. Other factors which may limit the generalizability of the findings include: the relatively small sample size for a national survey; the ethnically heterogeneous white population; non-fasting measures of HDL-cholesterol and total cholesterol; and moderately high proportions of missing data in the blood analytes, particularly for HbA1c. Furthermore, a larger sample would have allowed for more dose–response categories. Lastly, our accelerometer data are based on classifying horizontal movement intensity and cannot distinguish between different postures (i.e. sitting and standing).

## Conclusions

In conclusion, we demonstrate that in comparison to adults who are physically inactive with high sedentary time, those who are physically active have a more desirable health profile across multiple cardiometabolic markers even when combined with high sedentary time. In contrast, low sedentary time in the absence of physical activity is associated with higher HDL-cholesterol levels. By suggesting that being physically active may offset some of the deleterious consequences of a routinely sedentary lifestyle, this study further emphasises the importance of physical activity in the promotion and maintenance of health. However, given the observational design, the interaction and relative magnitude of effect of physical activity and sedentary behaviour on health needs further elucidation through experimental research in order to better inform public health policy and guidance.

## Availability of data and materials

Permission to use the 2008 Health Survey for England accelerometer data files can be obtained from the National Centre for Social Research (Natcen) (http://www.natcen.ac.uk/). All other data are openly available to download from the UK Data Archive (http://discover.ukdataservice.ac.uk/series/?sn=2000021).

## Additional file

Additional file 1: Figure S1. Categorical associations with markers of cardiometabolic health (beta coefficients (99 % CIs)). **Table S1** - Sensitivity analyses showing the weighted mutually exclusive behavioural category prevalence [*n*; %]. **Table S2** - Sensitivity analyses showing the categorical associations with markers of cardiometabolic health (beta coefficients (99 % CIs)). (DOCX 47 kb)
