# Supplementary material for: Associations of mutually exclusive categories of physical activity and sedentary time with markers of cardiometabolic health in English adults: a cross-sectional analysis of the Health Survey for England
Source: BMC Public Health. 2016 Jan 12;16:25. doi: 10.1186/s12889-016-2694-9 (PMC4709945; doi:10.1186/s12889-016-2694-9)
Supplement: Additional file 1: Figure S1. — Categorical associations with markers of cardiometabolic health (beta coefficients (99 % CIs)). Table S1 - Sensitivity analyses showing the weighted mutually exclusive behavioural category prevalence [n; %]. Table S2 - Sensitivity analyses showing the categorical associations with markers of cardiometabolic health (beta coefficients (99 % CIs)). (DOCX 47 kb) [file 12889_2016_2694_MOESM1_ESM.docx]

**Associations of mutually exclusive categories of physical activity and sedentary time with markers of cardiometabolic health in English adults: a cross-sectional analysis of the Health Survey for England**

**Authors:** Kishan Bakrania, Charlotte L. Edwardson, Danielle H. Bodicoat, Dale W. Esliger, Jason M.R. Gill, Aadil Kazi, Latha Velayudhan, Alan J. Sinclair, Naveed Sattar, Stuart J.H. Biddle, Kamlesh Khunti, Melanie Davies and Thomas Yates

**SUPPLEMENTARY MATERIAL**

Figure S1 - Categorical associations with markers of cardiometabolic health (beta coefficients (99% CIs))

Table S1 - Sensitivity analyses showing the weighted mutually exclusive behavioural category prevalence [n; %]

Table S2 - Sensitivity analyses showing the categorical associations with markers of cardiometabolic health (beta coefficients (99% CIs))

Figure S1 - Categorical associations with markers of cardiometabolic health (beta coefficients (99% CIs))

|      |
| --- |
| *‘Busy Bees’: Physically Active and Low Sedentary, ‘Sedentary Exercisers’: Physically Active and High Sedentary, ‘Light Movers’: Physically Inactive and Low Sedentary, ‘Couch Potatoes’: Physically Inactive and High Sedentary*  *All analyses accounted for primary sampling units, clustering and survey weights. Unadjusted and adjusted linear regression models were fitted for each cardiometabolic health marker with the ‘Couch Potatoes’ category selected as the reference group. The adjusted linear regression models controlled for: age; body mass index (except in the model with body mass index as the dependent variable); cardiovascular disease index; ethnicity; fruit and vegetable consumption; sex; smoking status; socioeconomic status; and accelerometer wear-time. Models with HDL-cholesterol and total cholesterol as the dependent variable were also controlled for both blood pressure medication and cholesterol medication. Similarly, the model with glycated haemoglobin as the dependent variable was controlled for any prescribed medication.* |

Table S1 - Sensitivity analyses showing the weighted mutually exclusive behavioural category prevalence [n; %]

| **Weighted Prevalence [n; %]** | | | | | |
| --- | --- | --- | --- | --- | --- |
| **Method** | **N** | **‘Busy Bees’** | **‘Sedentary Exercisers’** | **‘Light Movers’** | **‘Couch Potatoes’** |
| Reference | 2131 | 385; 18.6% | 743; 36.7% | 147; 6.8% | 856; 37.9% |
| 1 | 2131 | 385; 18.6% | 743; 36.7% | 147; 6.8% | 856; 37.9% |
| 2 | 2030 | 374; 18.9% | 707; 36.7% | 141; 6.8% | 808; 37.6% |
| 3 | 2131 | 495; 23.9% | 633; 31.4% | 215; 9.8% | 788; 34.9% |
| 4 | 2131 | 96; 4.6% | 241; 12.0% | 436; 20.8% | 1358; 62.6% |
| *‘Busy Bees’: Physically Active and Low Sedentary, ‘Sedentary Exercisers’: Physically Active and High Sedentary, ‘Light Movers’: Physically Inactive and Low Sedentary, ‘Couch Potatoes’: Physically Inactive and High Sedentary*  *Reference Method = mutually exclusive behavioural categories derived and utilised in the main analysis*  *Method 1 = missing data in the covariates were imputed using the behavioural category means (continuous variables: body mass index) and modes (categorical variables: smoking status and socioeconomic status)*  *Method 2 = participants with a cardiovascular disease index of ‘one or more cardiovascular diseases’ were excluded*  *Method 3 = ‘Low Sedentary’ was defined as residing in the lowest tertile of the ratio between the average sedentary time and the average light-intensity physical activity time*  *Method 4 = participants were only classified into the ‘physically active’ categories if they accumulated ≥150 minutes of moderate-to-vigorous physical activity per week in bouts of ≥10 minutes*  *All analyses accounted for primary sampling units, clustering and survey weights.* | | | | | |

Table S2 - Sensitivity analyses showing the categorical associations with markers of cardiometabolic health (beta coefficients (99% CIs))

| **Health Marker** | **Method** | **‘Busy Bees’** | **‘Sedentary Exercisers’** | **‘Light Movers’** | **‘Couch Potatoes’** |
| --- | --- | --- | --- | --- | --- |
|  |  | **Beta (99% CI)** | **Beta (99% CI)** | **Beta (99% CI)** |  |
| BMI  (kg/m^2^) | Reference | **-1.67 (-2.57, -0.77)** | **-1.64 (-2.43, -0.85)** | -0.66 (-1.92, 0.60) | Reference |
|  | 1 | **-1.61 (-2.51, -0.71)** | **-1.55 (-2.33, -0.78)** | -0.61 (-1.85, 0.64) | Reference |
|  | 2 | **-1.71 (-2.59, -0.84)** | **-1.67 (-2.48, -0.85)** | -0.62 (-1.93, 0.69) | Reference |
|  | 3 | **-1.70 (-2.63, -0.77)** | **-1.62 (-2.53, -0.71)** | -0.48 (-1.67, 0.71) | Reference |
|  | 4 | **-2.83 (-4.05, -1.62)** | **-2.00 (-1.20, 0.32)** | -0.44 (-1.20, 0.32) | Reference |
| Waist Circumference (cm) | Reference | **-1.17 (-2.28, -0.06)** | -0.71 (-1.56, 0.14) | -0.07 (-1.61, 1.47) | Reference |
|  | 1 | **-1.50 (-2.85, -0.15)** | -0.89 (-1.97, 0.20) | -0.67 (-2.42, 1.08) | Reference |
|  | 2 | **-1.19 (-2.32, -0.05)** | -0.66 (-1.55, 0.23) | -0.11 (-1.71, 1.50) | Reference |
|  | 3 | **-1.29 (-2.45, -0.14)** | -0.64 (-1.65, 0.38) | -0.29 (-1.74, 1.16) | Reference |
|  | 4 | **-1.83 (-3.61, -0.05)** | **-1.08 (-2.11, -0.05)** | -0.40 (-1.37, 0.56) | Reference |
| HDL-Cholesterol (mmol/L) | Reference | **0.09 (0.02, 0.16)** | **0.07 (0.02, 0.13)** | **0.11 (0.01, 0.21)** | Reference |
|  | 1 | **0.10 (0.04, 0.17)** | **0.08 (0.03, 0.14)** | **0.12 (0.02, 0.21)** | Reference |
|  | 2 | **0.09 (0.02, 0.17)** | **0.08 (0.02, 0.13)** | **0.10 (0.01, 0.20)** | Reference |
|  | 3 | **0.09 (0.02, 0.17)** | **0.08 (0.02, 0.15)** | **0.11 (0.03, 0.20)** | Reference |
|  | 4 | **0.20 (0.09, 0.31)** | 0.07 (-0.01, 0.15) | 0.04 (-0.02, 0.10) | Reference |
| Total Cholesterol (mmol/L) | Reference | 0.02 (-0.17, 0.22) | 0.17 (-0.01, 0.35) | 0.08 (-0.22, 0.38) | Reference |
|  | 1 | 0.02 (-0.17, 0.21) | 0.21 (-0.04, 0.37) | 0.09 (-0.20, 0.39) | Reference |
|  | 2 | 0.01 (-0.18, 0.21) | 0.18 (-0.01, 0.35) | 0.05 (-0.25, 0.35) | Reference |
|  | 3 | 0.02 (-0.19, 0.23) | 0.22 (-0.01, 0.45) | 0.11 (-0.19, 0.40) | Reference |
|  | 4 | -0.28 (-0.59, 0.02) | 0.04 (-0.19, 0.28) | 0.01 (-0.18, 0.19) | Reference |
| HbA1c  (%) | Reference | **-0.12 (-0.22, -0.01)** | **-0.11 (-0.23, -0.01)** | 0.26 (-0.11, 0.63) | Reference |
|  | 1 | **-0.13 (-0.23, -0.03)** | **-0.13 (-0.24, -0.02)** | 0.23 (-0.14, 0.59) | Reference |
|  | 2 | **-0.11 (-0.21, -0.01)** | -0.10 (-0.22, 0.02) | 0.28 (-0.11, 0.66) | Reference |
|  | 3 | **-0.11 (-0.23, -0.01)** | **-0.12 (-0.26, -0.02)** | 0.19 (-0.14, 0.52) | Reference |
|  | 4 | -0.07 (-0.18, 0.03) | **-0.12 (-0.23, -0.01)** | 0.05 (-0.07, 0.18) | Reference |
| *‘Busy Bees’: Physically Active and Low Sedentary, ‘Sedentary Exercisers’: Physically Active and High Sedentary, ‘Light Movers’: Physically Inactive and Low Sedentary, ‘Couch Potatoes’: Physically Inactive and High Sedentary*  *Reference Method = mutually exclusive behavioural categories derived and utilised in the main analysis*  *Method 1 = missing data in the covariates were imputed using the behavioural category means (continuous variables: body mass index) and modes (categorical variables: smoking status and socioeconomic status)*  *Method 2 = participants with a cardiovascular disease index of ‘one or more cardiovascular diseases’ were excluded*  *Method 3 = ‘Low Sedentary’ was defined as residing in the lowest tertile of the ratio between the average sedentary time and the average light-intensity physical activity time*  *Method 4 = participants were only classified into the ‘physically active’ categories if they accumulated ≥150 minutes of moderate-to-vigorous physical activity per week in bouts of ≥10 minutes*  *All analyses accounted for primary sampling units, clustering and survey weights. The adjusted linear regression models controlled for: age; body mass index (except in the model with body mass index as the dependent variable); cardiovascular disease index; ethnicity; fruit and vegetable consumption; sex; smoking status; socioeconomic status; and accelerometer wear-time. Models with HDL-cholesterol and total cholesterol as the dependent variable were also controlled for both blood pressure medication and cholesterol medication. Similarly, the models with glycated haemoglobin as the dependent variable were controlled for any prescribed medication.* ***Bold*** *indicates statistical significance at α = 0.01.* | | | | | |
